# Gun-Shot Wounds of the Hip—the Ball Passing around the Back

**Published:** 1845-08

**Authors:** 


*Gun Shot Wound in the Hip—the Ball passing around the Back.*—A person (W. A., residing in Southwark) led by curiosity to visit the scene of the riots in Kensington, whilst in the vicinity of the firing by the mob, received a musket ball in his left hip. He immediately fell, and was at once conveyed home by his friends. Dr. Condie was sent for, the messenger informing him on the road that Mr. A had been shot through the body. On arriving at the residence of the wounded man, the doctor was surprised to find neither in the countenance, pulse, or temperature of the patient's surface, any thing to indicate that he had just received so serious an injury. On examination, he found, directly over the great trochanter of the left femoris, a wound indicating the place of entrance of a ball, and on the left side, between the great trochanter and the anterior superior spinous process of the ilium, another wound where the ball had made its exit. The patient was able to rise up and support himself in the sitting posture without any difficulty or pain; and, on being requested, stood on his feet and walked some steps, with no other inconvenience than arose from a sense of constraint or stiffness at the hip joints; a feeling, as he expressed it, as though he was encompassed there with an iron band. The simplest dressings were applied, and a dose of Dover's powder administered to the patient. He rested well that night, and on the ensuing morning the nature of the injury he had received was rendered very evident by a broad red streak, extending from the two wounds around the back. The ball had passed, beneath the integuments, from its place of entrance, on the left side, across the posterior part of the body, to the right side, where it made its exit. The wounds were healed and the patient was entirely well within three weeks from the date of this accident.—*Transac. Coll. Phys. Phila.*

